# First detailed records of water mite larvae (Hydrachnidia: Hydrovolzidae, Hydryphantidae) parasitizing empidid flies (Diptera: Empididae: Clinocerinae)

**DOI:** 10.1016/j.ijppaw.2020.06.001

**Published:** 2020-06-11

**Authors:** Iwona Słowińska, Andrzej Zawal, Robert Stryjecki, Grzegorz Michoński

**Affiliations:** a University of Łódź, Department of Invertebrate Zoology and Hydrobiology, Banacha 12/16, 90-237, Łódź, Poland; b University of Szczecin, Institute of Marine and Environmental Science, Wąska 13, 71-415, Szczecin, Poland; c University of Life Sciences in Lublin, Department of Zoology and Animal Ecology, Akademicka 13, 20-950, Lublin, Poland

**Keywords:** Empididae, Clinocerinae, Acari, *Hydrovolzia*, *Panisopsis*, *Protzia*, Parasite

## Abstract

Five species of the subfamily Clinocerinae from the Tatra Mountains (S Poland) were observed to be parasitized by larval water mites. Two of them: *Kowarzia plectrum* Mik, 1880 and *Clinocera storchi* Mik, 1880 are recorded from this massif for the first time. In addition, *C. storchi,* is new for Polish fauna. The most infected species was *Clinocera appendiculata* Zetterstedt, 1838, following by *Wiedemannia mikiana* (Bezzi, 1899), *Clinocera storchi* Mik, 1880, *Kowarzia plectrum* Mik, 1880 and *Wiedemannia jazdzewski* Niesiołowski, 1987. The highest number of hosts occurred in the case of *Panisopsis curvifrons* (Walter, 1907) with five host species, following by both *Hydrovolzia placophora* (Monti, 1905) and *Protzia eximia* (Protz, 1896) with one species each. In the case of *Clinocera appendiculata* more parasites were recorded on males than on females and in *C. storchi* more parasites were recorded on females. The abdomen of the hosts was the most often chosen by water mites larvae.

## Highlights

• First detailed records of water mites parasitism on five Clinocerinae species.
• There are differences in infection of sexes in different host species.
• Abdomen was the most often chosen parts of the body by water mites larvae.
• The widest range of the body size occurred for *Panisopsis curvifrons* larvae.
• *Hydrovolzia placophora* is the species develop later than *Panisopsis curvifrons*.

## Graphical abstract

**Figure:**
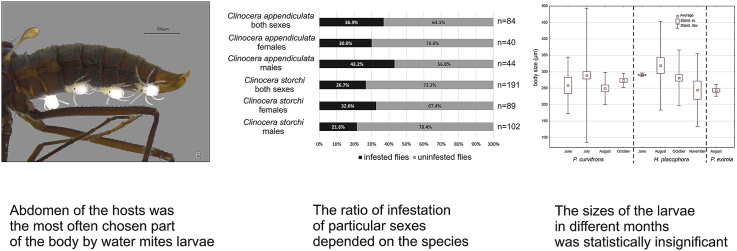

## 1 Introduction

The subfamily Clinocerinae (Diptera: Empididae) is a large group of predacious, long-legged flies that exist in lotic freshwater habitats. It contains 18 genera and well over 360 described species worldwide (Yang et al., 2007; Sinclair and Shamshev, 2014; Palaczyk et al., 2015). Adults are small to medium flies, usually greyish or sometimes brownish in colour, with generally narrow wings. So far as it is known, the immature stages of Clinocerinae are aquatic. Larvae live below the water surface predominantly in the moss or algal mats covering stones in the streams where they prey on small insects larvae, mainly on chironomids and simuliids (Vaillant, 1952, 1953, 1967). Sometimes larvae invade the pupal cocoons and attack the pupae (Zwick et al., 2011).

Among clinocerine genera, members of the genus *Clinocera* Meigen, 1803 usually occur in seepage habitats and also on wet stones or rocks and moss in headwater streams (Sinclair, 2008). In some cases they may be found in larger streams or rivers. In contrast, adults of *Wiedemannia* Zetterstedt, 1838) are generally confined to large and relatively clean, cool streams and rivers. Members of this genus are common resting on emergent stones and boulders. Adults of both genera prey on larvae and adults of chironomids and simuliids. In addition, adults of *Clinocera* are able to prey on larvae of thaumaleids as Sinclair (2008) reported. They are good and active fliers. However, most often, during the day, they remain on stones or boulders, on which they exhibit various forms of activity. On the other hand, during the night, they can gather in large numbers in the “sleeping association”, from where they can be easily caught using insect collecting nets (Wagner and Gathmann, 1996, first author observation). Members of the subfamily Clinocerinae are active mainly in spring and summer but they are often present during colder months.

It is well known that dipterans are one of the most important hosts for larvae of many families of water mites (Acariformes: Hydrachnidia). Members of 17 families of nematoceran and five brachyceran flies are known as hosts for water mites (Martin and Gerecke, 2009). The family Chironomidae is most frequently parasitized by larvae of water mites (Smith and Oliver, 1986; Smith et al., 2001). Information of the interactions between empidid flies and mites is sparse. In particular, there is little data available on parasitism on the adults of the subfamily Clinocerinae (Tuzovskij et al., 2001; Sinclair, 2008).

In Poland, the subfamily Clinocerinae has been relatively well studied, especially in mountainous areas (Niesiołowski, 1990, 2005; Klasa et al., 2000; Palaczyk and Klasa, 2003; Krysiak and Niesiołowski, 2004; Krysiak, 2005; Palaczyk and Słowińska-Krysiak, 2013; Słowińska-Krysiak, 2014a, b; Słowińska, 2017, 2019). It is represented by 38 species, including *Bergenstammia glacialis* Palaczyk et Słowińska (2015), the species described recently from the Tatra Mountains (Palaczyk et al., 2015). Most species belonging to this subfamily occur in the mountains, however, many areas in the Sudetes and the Polish Carpathians are scarcely investigated. Despite undertaking long-term field studies on the family Empididae by the abovementioned authors, no cases of mites parasitizing on the Clinocerinae species have been reported in the country. Our study provides the first detailed records of water mites parasitic on Clinocerinae species from Poland.

## 2 Material and methods

**Fig. 1:**
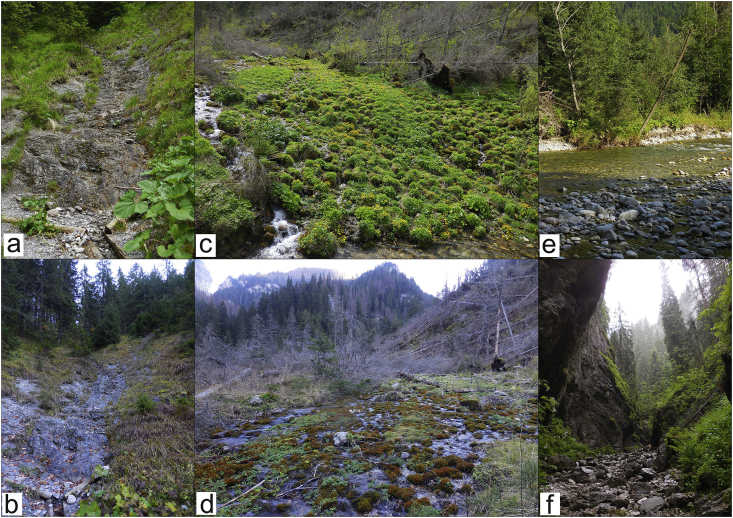
Types of habitat where infested and uninfested clinocerine species were found; a – the Mała Łąka Valley, June 2017; b – the same locality, October 2017; c – the Kościeliska Valley, May 2018; d – the same locality, November 2018; e – Białka River, Łysa Polana, August 2017; f – Cracow Gorge, June 2017 (photographs by I. Słowińska).

Material analysed in this paper was collected during the regular studies on the Clinecerinae and Hydrachnidia in the area of Tatra mountains, during the summer-autumn period (from May to November in 2015, 2017 and 2018 years). The research was carried out at 75 sampling sites in the High Tatras (built of granites with typical alpine-type glacial relief) and the Eastern Tatras (built of metamorphic, crystalline and sedimentary rocks). Infested clinocerine adults were caught with an insect net in only four of the 75 sampling sites (Fig. 1):

1. The Mała Łąka Valley, a small couloir, 980 m–49°16′15″ N; 19°54′03″ E;
2. The Kościeliska Valley, a series of karst springs, which form a lamellar inundation, 1030 m–49°14′29″ N; 19°51′50″ E;
3. Cracow Gorge, an intermittent stream, 1100 m–49°14′20″ N; 19°51′59″ E;
4. Łysa Polana (Clearing), Białka River, 970 m–49°15′55″ N; 20°07′01″ E.

Flies were preserved in 95% ethanol on-site. Larvae of water mites were removed from clinocerines by using forceps after recording their number and attachment sites in different regions of the host body. The larvae were mounted by embedding in the Hoyers’ medium, and identified by using the keys and original papers of Tuzovskij et al. (2001) and Martin (2000, 2003). Adults of the genus *Clinocera* were determined by reference to the original description (Mik, 1880) and using a key by Sinclair (1999). Photographs were taken using a Leica M205C stereomicroscope. Material was deposited in the Department of Invertebrate Zoology and Hydrobiology of the University of Łódź (Łódź, Poland).

Prevalence and intensity of infestation (number parasites per infested host individual) relative to hosts, sexes of hosts and parts of host body were analysed. One-way ANOVA was used, based on the log-transformed dataset, to assess the statistical significance of differences between number of water mite larvae on different host's sexes and particular parts of host's body, their size of body and number of hosts, and Shapiro-Wilk test showed that all log-transformed dataset were normally distributed (Jayaraman, 1999). These analyses were performed using the Statistica 13.1 PL software.

## 3 Results

**Fig. 2:**
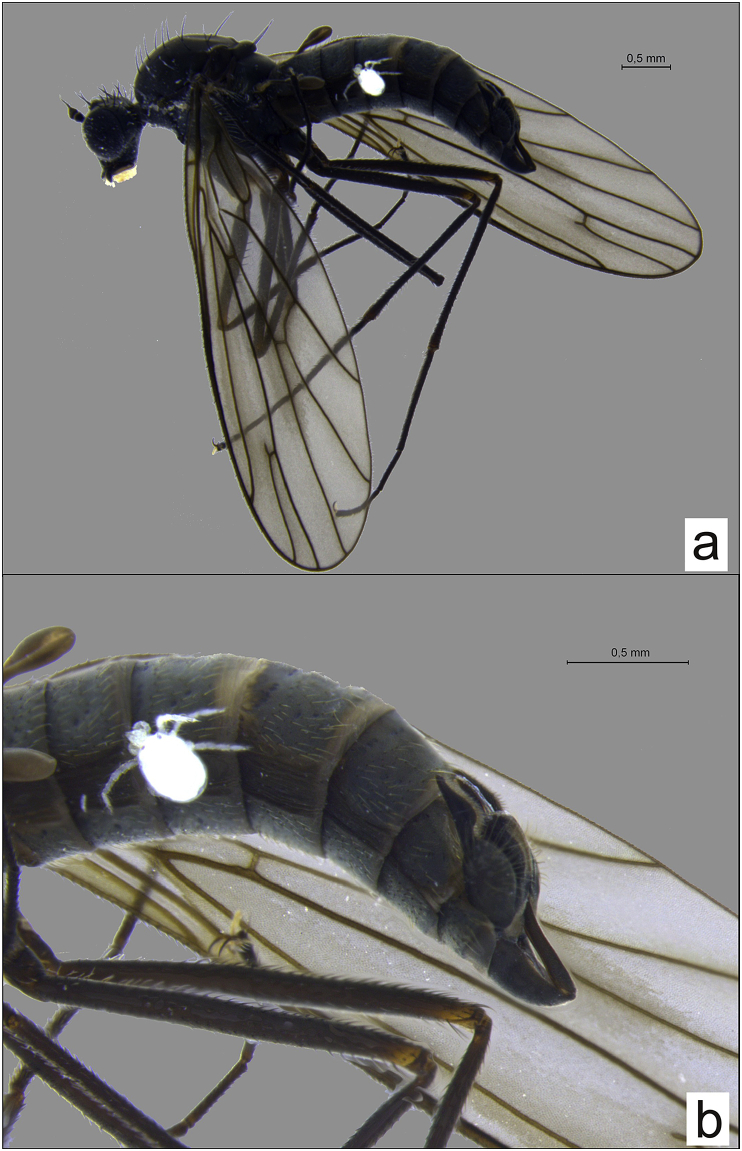
*Clinocera appendiculata* parasitized by *Panisopsis curvifrons*; a – habitus, b – abdomen.

**Fig. 3:**
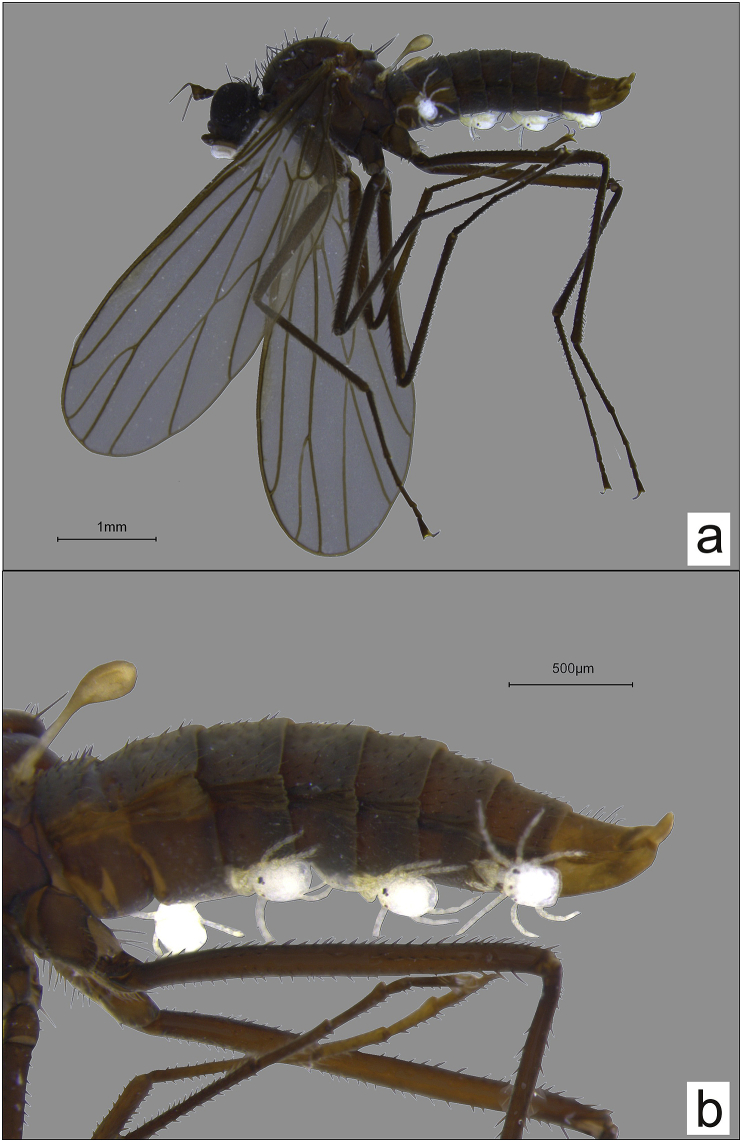
*Wiedemannia mikiana* parasitized by *Panisopsis curvifrons*; a – habitus, b – abdomen.

**Fig. 4:**
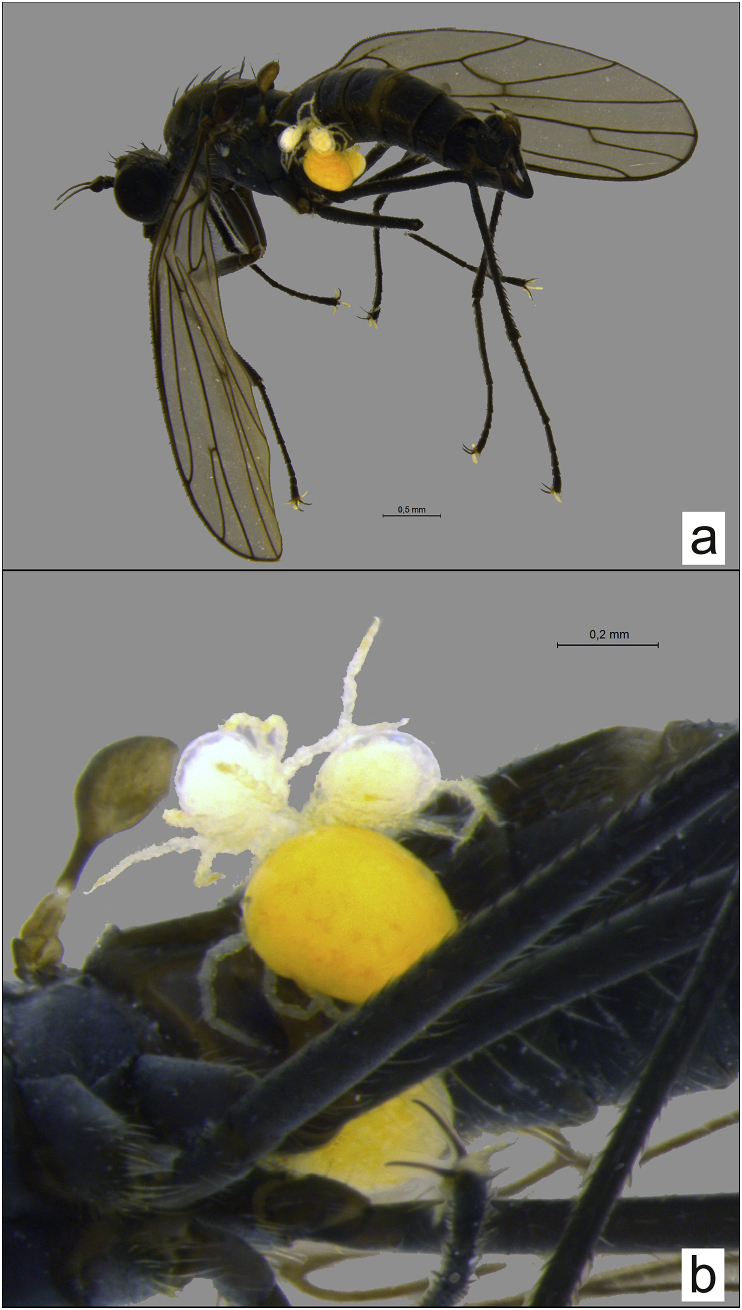
*Clinocera storchi* parasitized by *Panisopsis curvifrons*; a – habitus, b – abdomen.

Over the three year period a total of 315 individuals (171♂♂, 144♀♀) of Clinocerinae were collected. Among them, 91 individuals (44♂♂ and 47♀♀) representing five species were infested with larval water mites (Table 1). We larvae represented three species in two families: *Hydrovolzia placophora* (Monti, 1905) (Hydrovolziidae), *Panisopsis curvifrons* (Walter, 1907) and *Protzia eximia* (Protz, 1896) (both from Hydryphantidae). Three of the five species were previously known from the Tatras: *Clinocera appendiculata* Zetterstedt, 1838, *Wiedemannia jazdzewskii* Niesiołowski, 1990 and *W. mikiana* (Bezzi, 1899) (Niesiołowski, 1990). Of the two remaining species, *Kowarzia plectrum* Mik (1880), is new for the Tatras, and *Clinocera storchi* Mik (1880), is new for Polish fauna. Infested adults were collected in June, July, August, October and November in 2015, 2017 and 2018. The clinocerine species differed significantly in mean number of host individuals that had at least one mite (F = 7.037; df = 5; p < 0.01 0.003516). The most infected species was *Clinocera appendiculata* (Fig. 2a and b), following by *Wiedemannia mikiana* (Fig. 3a and b), *Clinocera storchi* (Fig. 4a and b), *Kowarzia plectrum* and *Wiedemannia jazdzewski*. *Clinocera appendiculata* and *Wiedemannia mikiana* were infested by 2 species of water mite, and *Clinocera storchi*, *Kowarzia plectrum* and *Wiedemannia jazdzewski* by one parasitic species. The highest number of hosts occurred in the case of *Panisopsis curvifrons* with five, following by *Hydrovolzia placophora* and *Protzia eximia* with one host species (Table 1).

**Table 1:** Occurrence of larvae of water mites recorded on particular host species.

Host	Parasite					
	Panisopsis curvifrons		Hydrovolzia placophora		Protzia eximia	
	prevalence %	intensity	prevalence %	intensity	prevalence %	intensity
*Clinocera storchi*	27.1	1–5	–	–	–	–
*Clinocera appendiculata*	26.1	1–3	43.8	1–3	–	–
*Kowarzia plectrum*	23.1	1–2	–	–	–	–
*Wiedemannia mikiana*	33.3	4	–	–	6.1	1
*Wiedemannia jazdzewski*	1	1	–	–	–	–

**Fig. 5:**
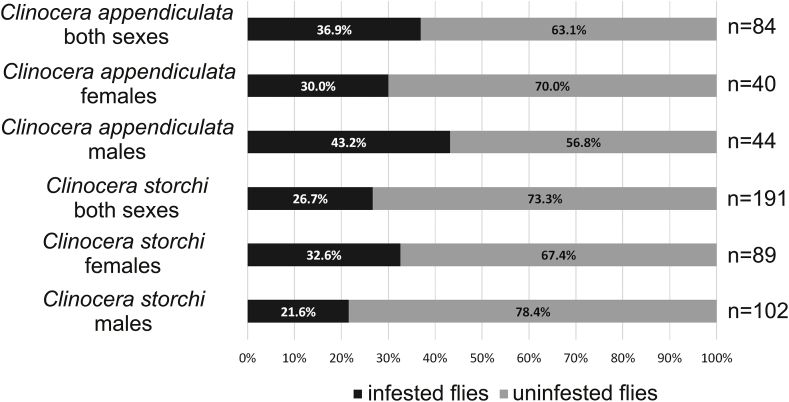
Percentage of mite-infested and uninfested *Clinocera appendiculata* and *C. storchi*.

Female flies were more infected than males (Table 2, Fig. 5), differences between number of infected hosts was statistically significant (F = 11.881; df = 1; p < 0.01), and differences between number of parasites was statistically significant (F = 12.942; df = 1; p < 0.01). The same differences between host sexes were in the case of parasitizing by *Panisopsis curvifrons* where female were more infected, and *Hydrovolzia placophora* where males were more infected: differences between number of infected hosts was respectively statistically significant (F = 8.521; df = 1; p < 0.02), (F = 11.21305; df = 1; p < 0.03), and differences between number of parasites were statistically significant (F = 12.942; df = 1; p < 0.01), (F = 15.13395; df = 1; p < 0.01). In the case of *Clinocera appendiculata* more parasites were recorded on males than on females (Table 2, Fig. 5), and the differences was statistically significant (F = 9.785; df = 1; p < 0.02). In the case of *C. storchi* more parasites were recorded on females than on males (Table 2, Fig. 5), but the differences was statistically insignificant (F = 0.013; df = 1; p > 0.05).

**Table 2:** Occurrence of water mite species on sexes of hosts: number of parasites/prevalence/intensity.

Host	Parasite							
	*Panisopsis curvifrons*		*Hydrovolzia placophora*		*Protzia eximia*		Number of: parasites/hosts	
	male	female	male	female	male	female	male	female
*Clinocera storchi*	30/21.1/1-4	42/34.1/1-5					30/20	42/28
*Clinocera appendiculata*	11/44.4/1-3	3/14.3/1	21/50.0/1-4	8/33.3/1-3			32/24	21/13
*Kowarzia plectrum*	2/20.0/1-2	1/33.3/1					2/2	1/1
*Wiedemannia mikiana*		4/50.0/5			1/5.5/1	2/6.5/1	1/1	6/3
*Wiedemannia jazdzewski*		2/13.2/1						2/2
Number of: parasites/hosts	43/26	52/35	21/12	8/10	1/1	2/2	65/47	72/47

Water mite larvae were more frequently attached to the host's abdomen than to the head, cervix, thorax or legs (F = 5.44608; df = 1; p < 0.05), but intensity of infestation was the same on each part of body (F = 0.54930; df = 1; p > 0.05). The situation was the same both for all species of hosts (Table 3), but larvae of *Hydrovalzia placophora* were more commonly found on the thorax and legs (Table 4).

**Table 3:** Place of attaching of parasites on the host body in particular host species: total number of larvae/maximum number of the larvae on one host/average number of larvae on one host.

Host	Part of body						
	head	neck	thorax	I legs	II legs	III legs	abdomen
*Clinocera storchi*	1	1	1	5/2/1.25		2/1	59/3/1.18
*Clinocera appendiculata*			8/3/3	14/3/1.14	4/1	9/2/1.05	12/3/1.1
*Kowarzia plectrum*				1			2/1
*Wiedemannia mikiana*				1			6/4/2
*Wiedemannia jazdzewski*							2/1

**Table 4:** Place of attaching of particular species of parasites on the host body: total number of larvae/maximum number of the larvae on one host/average number of larvae on one host.

Parasite	Part of host's body						
	head	neck	thorax	I legs	II legs	III legs	abdomen
*Panisopsis curvifrons*	1	1	1	6/2/1,2		2/1	78/4/1,15
*Hydrovolzia placophora*			8/3/3	14/3/1.14	4/1	9/2/1.05	
*Protzia eximia*				1			2/1

**Fig. 6:**
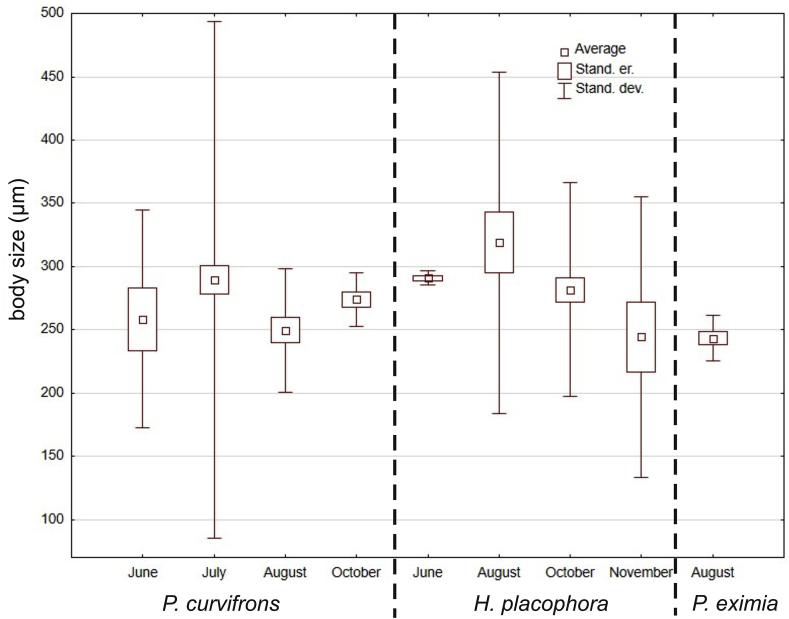
Dependence of body size of parasites on the period of occurrence.

Infected clinocerids were recorded in June, July, August, October and November, and the most number of parasites occurred in July (Table 5), differences between number of parasites was statistically significant (F = 2.83428; df = 4; p < 0.05). Larvae of *Panisopsis curvifrons* were most abundant in July, *Hydrovolzia placophora* in October and *Protzia eximia* in August. Larvae of *Panisopsis curvifrons* were recorded from June to October, *Hydrovolzia placophora* from June to November, and *Protzia eximia* only in August (Table 5, Fig. 1). The body size of water mite larvae varied from 182 to 719 μm. The widest range of the values occurred for *Panisopsis curvifrons* 182–719 μm, follow by *Hydrovolzia placophora* 200–396 μm and *Protzia eximia* 233–250 μm (Fig. 6). The sizes of the larvae in different months was statistically insignificant (F = 0.29; df = 4; p > 0.05).

**Table 5:** Occurrence of particular parasite species in different months.

parasite species	*Panisopsis curvifrons*				*Hydrovolzia placophora*				*Protzia eximia*
month	June	July	August	October	June	August	October	November	August
number of larvae/prevalence/intensity	3/18.8/1	83/32.6/1-5	6/1.25/1-4	3/11.7/2	2/25/2	8/100/1-3	19/33.3/1-4	4/25/2	3/6.1/1

## 4 Discussion

Empididae have seldom been reported as hosts for water mite larvae (Smith and Oliver, 1986). Up to now only *Hydrovolzia cancellata* Walter, 1906 and *H. placophora* have been found on the family. *Hydrovalzia placophora* was found on *Clinocera* and *Roederioides* genera, whereas *H. cancellata* has been reported from *Clinocera*, *Wiedemannia* and *Kowarzia* (Tuzovskij et al., 2001). Our study adds two other water mite species (*Panisopsis curvifrons* and *Protzia eximia*) to the list of parasites of family Empididae. *Hydrovolzia placophora* and *Protzia eximia* are opportunistic and known as parasites of other hosts, while *Panisopsis curvifrons* was recorded only from Chironomidae (Smith and Oliver, 1986; Tuzovskij et al., 2001; Gerecke and Martin, 2006).

The most infected clinocerids belongs to the most abundant species (*Clinocera appendiculata*, *C. storchi*, *Wiedemannia mikiana*) which is common for other water mite species, and intensity of infestation is rather low, as usual in dipterans and in contrast with odonates, beetles and bugs. Earlier literature data pointed to a much broader list of host species for *Hydrovolzia placophora* and *Protzia eximia* than for *Panisopsis curvifrons* (Smith and Oliver, 1986; Tuzovskij et al., 2001; Gerecke and Martin, 2006). Our research does not confirm this pattern as *Panisopsis curvifrons* was recorded on five hosts species, and *Hydrovolzia placophora* and *Protzia eximia* only on one host species each. It seems that the later two species of parasites occur on more orders of insects while *Panisopsis curvifrons* attach only dipterans but at least of three families (Simulidae, Chironomidae and Empididae). Among of the three water mite species *Hydrovolzia placophora* and *Panisopsis curvifrons* are crenobionts, and *Protzia eximia* has a little wider distribution including springs and streams. This is reflected in the lists of host species, where *Hydrovolzia placophora* has a wide diversity of semiaquatic or semiterrestial host species, *Panisopsis curvifrons* parasitized on simuliids, chironomids and empidids occurred in springs, and *Protzia eximia* has the widest list of typically aquatic host species including Diptera and Trichoptera (Davids et al., 2007; Di Sabatino et al., 2010).

In water mite literature there are many reports assessing differential parasitism of host sexes. Some of them indicate differences in infestation of different sexes, others indicate the lack of such differences. For example Atwa et al. (2017), Kirkhoff et al. (2013) and Edwards and Smith (2003) show females of mosquitos and chironomids were more infected than males, but Martin and Stur (2006) did not see such differences between sexes in Limoniidae, Simuliidae, Dixidae and Chironomidae. Differing reports of sex-biased parasitism also apply to odonates. Zawal (2004), Baker et al. (2008) presented data suggesting clear preferences in relation to the infestation of females in *Coenagrion puella* and *C. pulchellum*. However, they were not confirmed in other papers (Zawal, 2006a, b). Ilvonen et al. (2016) found significant differences between prevalence and intensity of infestation the sexes within certain host species of Odonata, but on a general level of Odonata more parasitized sex does not exist in the order. It seems that the situation is quite complicated and the differences in sex infestation depends on the host species and the parasite species. In our research *Panisopsis curvifrons* preferred famales, and *Hydrovolzia placophora* preferred males as a host, but the reason are unknown.

Usually larvae of water mites attach to the thorax or abdomen of their hosts; this holds for most dipterans, odonates, water beetles and water bugs (Biesiadka and Cichocka, 1994; Zawal, 2002, 2003; Baker et al., 2007; Stryjecki et al., 2015). This is unsurprising given that the thorax and abdomen make up the majority of the host's body. Additionally the integument is soft on abdomen, and in the case of beetles and bugs, water mite larvae are protected by elytra and hemelytra (Biesiadka and Cichocka, 1994; Zawal, 2002, 2003; Arjomandi et al., 2019). Most probably legs play the same role as elytra and enclose the water mite larvae attached to the thorax (Odonata, Diptera) (Zawal and Buczyński, 2013). In a few cases water mite larvae attached to the legs of their hosts: *Ranatra linearis* (Linnaeus, 1758) parasitized by *Hydrachna gallica* Thor, 1916 (Zawal et al., 2013), and Clinocerinae parasitized by *Hydrovolzia cancellata* (Tuzovskij et al., 2001). It is not known whether they attack soft hosts shortly after the moulting, or whether they are hard integuments of fully mature forms.

The most numerous occurrence and the highest activity (including procreation activity) water mites of crenophilous and crenobionts species coincides with the later period of the year (late summer or autumn) than limnophilous species (Zawal, 2002, 2003; Martin and Stur, 2006; Zawal and Szlauer-Łukaszewska, 2012). Among the investigated species *Hydrovolzia placophora* has the most activity period annually later than *Panisopsis curvifrons.*

The body size range of all three water mite species larvae was very wide in all months, that means the time of ovipositions is elongated. And body size of the larvae was much smaller than body size of the adults (Di Sabatino et al., 2010), which means that significant body gains were realized in the deutonymph stage like in other water mite larvae parasitizing on Diptera and Trichoptera (Martin and Stur, 2006; Buczyńska et al., 2015) unlike the water mite larvae parasitizing on odonates, water beetles and water bugs (Biesiadka and Cichocka, 1994; Zawal, 2002, 2003; Zawal et al., 2017).

## Funding

This research did not receive any specific grant from funding agencies in the public, commercial, or not-for-profit sectors. The study was supported only by both the statutory funds of the University of Szczecin and University of Łódź.

## Availability of data and material

The datasets generated during and/or analysed during the current study are available from the corresponding author on reasonable request.

## Declarations of competing interest

There is no conflict of interest.
